# Crossing the Line: Selection and Evolution of Virulence Traits

**DOI:** 10.1371/journal.ppat.0020042

**Published:** 2006-05-26

**Authors:** Nat F Brown, Mark E Wickham, Brian K Coombes, B. Brett Finlay

## Abstract

The evolution of pathogens presents a paradox. Pathogenic species are often absolutely dependent on their host species for their propagation through evolutionary time, yet the pathogenic lifestyle requires that the host be damaged during this dependence. It is clear that pathogenic strategies are successful in evolutionary terms because a diverse array of pathogens exists in nature. Pathogens also evolve using a broad range of molecular mechanisms to acquire and modulate existing virulence traits in order to achieve this success. Detailing the benefit of enhanced selection derived through virulence and understanding the mechanisms through which virulence evolves are important to understanding the natural world and both have implications for human health.

## Introduction

The Earth provides an environment with considerable heterogeneity. Commensurate with this, the Earth is inhabited by an outstanding array of species, varying significantly in complexity and design. The vast majority of these species are part of an intricate network involving direct and indirect interactions that constitute the global ecosystem. Highlighting the span of this network, the environmental niches occupied by some species extend to niches present either on or within other species. These interactions generally fall into categories such as symbiosis/mutualism, commensalism, or parasitism. The species participating in these direct interactions range from viruses to metazoans, act in various capacities, and in some instances involve three or more species. It may be useful to consider that the categorisation of these direct interspecies interactions as mutualism, commensalism, or parasitism represents a qualitative simplification.

In addition to their intrinsic interest, instances where humans act as host in parasitic interactions are significant for human health. Throughout history, humanity has been afflicted by many of the infectious diseases that we refer to here. More recently, improved public health and therapeutic interventions have decreased the impact of infectious diseases on human health. Nonetheless, the significance of infectious diseases as a cause of human mortality is underscored by World Health Organization statistics for the year 2002, which state that infectious diseases resulted in more years-of-life-lost than all non-communicable diseases across the globe. The impact of infectious disease on human health is widely considered to be increasing, in part through failure of previously efficacious therapeutics. This in itself is a prime demonstration of how species can evolve to fill an empty environmental niche. The motivation to understand how parasitic relationships are maintained is obvious given the toll infectious diseases take on human health. However, the intellectual approach taken to study parasitism is perhaps most effective when an anthropocentric view is rejected in favour of an ecological view [1].

Parasitism is merely one example of how species exist in nature. It exemplifies the capacity of organisms to evolve forms able to fill different environmental niches. All species have resulted from a “struggle for existence” [2]. As originally postulated, this theory would lead us to believe that species have been shaped through competition to exist in particular environmental contexts. Pathogens exist in this same evolutionary framework, and in this review we will discuss the selective pressures applied to pathogens and the evolutionary mechanisms used by pathogens to overcome these pressures. In the literature to date, each of these topics is generally discussed with little consideration for the other. By considering selection and mechanisms of evolution together, we hope to provide an appropriate contextual starting point for an all-encompassing view of pathogen evolution.

## Definitions

It will be useful to the reader to understand our definitions of *virulence* and *pathogen* because various definitions of these terms are used in the field of infectious disease and pathogen biology. It is not, however, our intention to redefine these terms for the field.

### 

#### Virulence.

Definitions of virulence typically relate to the capacity of a pathogen to cause damage or disease in the host [3,4]. Essentially this is related to the cost to the host. A frequent measure of virulence is the mortality rate associated with infection from a pathogen, although it is important to consider alternative measures in some circumstances [4–11]. For the purpose of this article, we suggest a slight modification to the current definition of virulence. Virulence should be defined as the damage to the host during infection with a pathogen. This definition provides clarity in the many instances where pathogens themselves do not actively cause a great deal of damage, rather the damage is caused by the host response. Despite common discussion of pathogenicity and virulence as defining slightly different characteristics [3], we consider this to be unclear and argue that pathogenicity and virulence are synonymous.

#### Pathogen.

The definition of pathogen has been discussed in detail with some degree of disagreement [3,12]. We define a pathogen as an organism capable of colonising a host organism where the interaction results in disease. Consequently for the purpose of this review, it is the diseased host that is integral to the definition of pathogen. Casadevall and Pirofski had concern that defining a pathogen as an organism that causes disease in a host was inadequate because some pathogens don't cause disease in all hosts [3]. This concern is unnecessary when one considers that hosts differ, both within the same species, and especially between different species. For example, Pseudomonas aeruginosa causes disease in individuals with burns or cystic fibrosis but does not cause disease in healthy individuals.

Koch's postulates either cannot be ethically tested in the case of human-specific pathogens, or care must be taken to select the appropriate host to test the virulence of a pathogen that affects other hosts. This is frequently a challenge for the experimentalist and Koch's postulates cannot be applied to provide a categorisation of a species as being pathogenic.

The conditional capacity for pathogens to be involved in interactions that result in disease has led to the use of adjectives to help describe the circumstances where a pathogen is associated with disease. A commonly used adjective is *opportunistic*. This is typically applied to pathogens that cause disease only in hosts that are immunocompromised or whose pathogenesis is facilitated by traumatic breaching of an epithelial barrier. These organisms are frequently commensal, i.e., colonise a ‘normal' host with no detrimental outcome for the host [12]. A less commonly used adjective is *accidental*. This refers to pathogens that are adapted to one particular host, in either a parasitic, commensal, or symbiotic relationship, but when infecting a host that the pathogen does not ordinarily encounter, a disease results [13]. For example, zoonotic pathogens can be considered accidental because they have usually not adapted to the accidental host in a manner allowing efficient transmission. It is apparent that some of the terminology used in the field of infectious disease research is unclear. Recent attempts to clarify this situation have been inadequate for contemporary research [3,14], and it would perhaps be useful for this matter to be revisited in a work focussed on developing a unifying lexicon for use in scientific discourse on pathogens.

## Selection for Virulence

**Figure 1:**
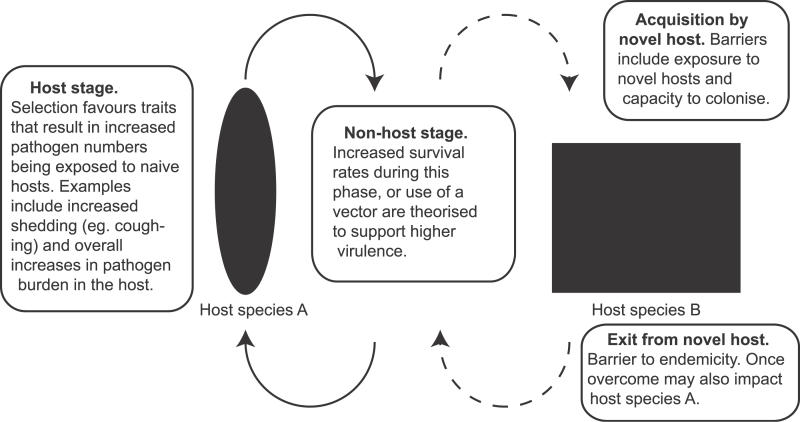
Flow Diagram of a Generic Pathogen Life Cycle Indicating the Selective Pressures That Have Been Suggested or Shown to Be Important in Shaping Pathogen Virulence The filled ellipse represents host species A (the major host), and the filled rectangle represents host species B (the novel host).

When discussing how virulence traits benefit pathogens, it is important to consider the pressures applied during the entire life cycle of the pathogen (Figure 1). The pathogen life cycle can vary considerably, from complex multiple-host life cycles of many helminths to direct host-to-host life cycles of sexually transmitted pathogens. Additionally, certain pathogens such as the bacterial species *Vibrio cholerae,* exist successfully in a non-host aquatic environment [15]. In simple terms, the evolutionary success of most pathogens requires efficient iteration of its life cycle. How the virulence of a pathogen affects this success is discussed below.

The existence of pathogens has often been viewed throughout history as being a transient step during evolution to a commensal state. The argument for this frequently states that parasitic interactions are poorly adapted interactions that are relatively young in evolutionary terms. Following from this, as time proceeds the pathogen is hypothesised to evolve to a commensal state through progressively decreased virulence [16,17]. This wholesale view is now widely disregarded. Typically, in instances of direct interactions between species, simple boundaries can be drawn to distinguish commensal behaviours from parasitic behaviours. By and large, commensal behaviour does not involve colonisation of privileged sites in the host and hence does not elicit a strong inflammatory response.

In contrast, parasitic behaviour most often involves colonisation of privileged sites and, as such, the host responds with a vigorous inflammatory response. Pathogens always display parasitic behaviour, and may in addition display commensal behaviour. Therefore, pathogens and commensals do not compete in most instances except where an epithelial surface must be colonised during initial stages of pathogenesis. Many pathogens do not display any behaviours associated with commensalism, hence evolution to a commensal state would typically involve adaptation to an entirely different environment within a host as well as the development of strategies to allow success when competing against the microbiota at that site. This would have to occur rapidly in order to provide substantive benefit to support selection of such traits. Whilst this is possible, a more likely occurrence would be an increase in the efficiency of the pathogenic strategy that would be selected at the expense of the less efficient progenitor. In the case of opportunistic pathogens, which clearly can display commensal and parasitic behaviours, it is more difficult to hypothesise. Many opportunistic pathogens appear to rely more on commensalism for survival. With virulence strategies playing a largely unknown role in the propagation of these species (see below), the role for virulence in the survival of these species is largely unknown. Clearly, if virulence strategies were important to the propagation of an opportunistic pathogen, an increase in the efficiency of these strategies would be expected to enhance the success of the species.

In understanding how virulence might be selected, it is also necessary to understand the implications of virulence mechanisms during the infectious stage of the infection. The symptoms associated with certain diseases may directly facilitate the spread of the pathogen; for example, respiratory infections that result in coughing and sneezing [18]. This sign of infection is the result of pathogen virulence mechanisms and is hypothesised to increase the output of the pathogen for subsequent infection of new hosts. Hence, higher efficiency of such virulence mechanisms would be expected to out-compete lower efficiency, as it would increase the output of the pathogen for transmission. However, there are numerous virulence strategies that do not appear to provide an obvious selectable advantage, such as when pathogens replicate at sites of the host from where the pathogen is not transmitted [11]. Many examples of this are opportunistic pathogens, which have both commensal and parasitic traits where the commensal colonisation of an epithelial surface can account for the spread of such species between hosts, and hence the existence of such species. Nonetheless, the question remains, how are the virulence traits of these organisms positively selected? In such instances, a virulence factor would be selected if it were beneficial at other important stages of the pathogen life cycle, including non-host stages. The benefit granted to clones at non-transmissible sites in the host would be short-term and a dead end. Alternatively, the pathogen populations at sites in the host thought to be unimportant for transmission to new hosts may actually form a reservoir for traffic to sites of the host that are competent for spread. A better understanding of the biology of these relationships will likely provide more rational hypotheses.

If virulence is positively selected as suggested by most modern theorists, this suggests that a relationship exists between virulence and pathogen load in the host. How closely related is virulence to the ability to replicate to relatively large numbers within a host? It is important to consider the ability to replicate as being more complex than a simple reflection of the efficiency of generic, core metabolism. Many pathogen-specific processes are involved, including, for example, evasion of the immune system. It would be expected that if a given pathogen acquired an adaptation allowing growth to higher numbers in the host, the cost to the host would increase, assuming the unit of damage to the host per unit of pathogen was unchanged. This includes damage mediated through active pathogen processes, damage mediated through host responses, and the additional nutrient burden of supporting the higher load of pathogens. In many host–pathogen systems this logic holds true, high pathogen burden indeed positively correlates with virulence, though this is often not directly discussed [4,17,19]. For this to translate into a selectable advantage in the longer term, it must increase the efficiency of the pathogen life cycle as a whole in a sustainable manner.

An important factor that is theorised to modulate the virulence of a successful pathogen is the mode of transmission. A common comparison is the virulence of vector-borne pathogens versus directly transmitted pathogens. It has been hypothesised that vector-borne pathogens allow for the selection of higher virulence levels than those that are directly transmitted [20,21]. This hypothesis assumes that increased virulence is associated with a reduction in normal host behaviours and mobility [21]. The higher virulence of vector-borne pathogens is afforded by transmission being less dependent on host mobility, and may even be enhanced by a reduction in host mobility increasing exposure to vectors. In contrast, direct transmission commonly requires active host behaviours to expose naive hosts to the pathogen. An alternative comparison is between horizontal and vertical transmission. While vertical transmission is not infectious, there are various direct interactions between species where transmission is strictly vertical. These pathogens rely on infected-host reproduction to propagate themselves, and as such the success of such pathogens is linked to the evolutionary success of infected hosts. It is predicted that vertically transmitted pathogens should evolve towards lower virulence, allowing greater success of their hosts. Using barley stripe mosaic virus infection of *Hordeum vulgare,* it has been found in that vertical transmission evolves towards decreased virulence whilst horizontal transmission evolves towards increased virulence [22].

A concept related to the association of virulence with mode of transmission is the capacity of the pathogen to survive in the external environment [16]. In a retrospective study of various human respiratory pathogens, a positive correlation between environmental (non-host) survival time and pathogen virulence was obtained [7]. This suggests that species capable of long-term environmental survival are less dependent on direct infected-host-to-uninfected-host contact for transmission and are able to evolve to higher relative virulence levels. Similar to the hypothesis for vector-borne pathogens, as a pathogen becomes less dependent on normal host function for transmission, a higher pathogen burden is positively selected, and this correlates with increases in virulence. A general strategy of horizontally transmitted pathogens during infection would be to produce the largest number of infectious particles per infected host phase. For pathogens with long environmental survival times, this may be achieved in a short period of time by reaching very high pathogen burdens during infection and waiting out a longer non-host phase for subsequent infections. An example of a bacterial pathogen using high virulence/long extra-host survival strategy would be *Bacillus anthracis,* which is extremely virulent and produces durable, long-lived endospores during non-host phases [7]. Conversely, pathogens with short extra-host survival times would rely on closer contact between hosts, at least in a temporal sense. This would necessitate relatively normal host function and a longer duration of infection, hence lower virulence, where the chances of productive host–host contacts would be maximised. Mycoplasma pneumoniae represents a good example of this strategy, typically causing a mild disease commonly referred to as walking pneumonia and having a short-lived and sensitive non-host phase [7].

Much of the above discussion on selection refers exclusively to species that are adapted to a particular host species. What of the selective pressures applied to accidental pathogens as they forge new ground in a previously unencountered host species? This may be quite common and therefore should be considered an important aspect of pathogen evolution. The most restrictive barriers to extending the host range of a pathogen are exposure to other host species, obviously the ability to colonise by the pathogen must be efficiently transmitted to subsequent hosts. Establishment in a new host species depends on numerous variables linked to the susceptible host population size and density, infection dynamics relating virulence to the spread of the pathogen and so forth [23]. The pathogen may become endemic, epidemic, or somewhere in between. Newly adapted pathogens that cause epidemics may become extinct, as occurred with the 1918 pandemic strain of influenza virus [24]. Once established with a degree of endemicity, pathogen virulence would then be subject to the selective pressures discussed above, where sibling clones compete with each other for natural selection.

## Evolution of Virulence

While it is indeed important to consider the entire life cycle of pathogens, it is the nature of their interaction with hosts that defines them as pathogens; consequently, this interaction has provided immense selection pressure for pathogen (and host) evolution. Invertebrate and vertebrate hosts possess an innate immune system to deal with pathogens; however, this system lacks specificity. Vertebrates possess the additional and superior protection of an adaptive immune response that has memory over individuals' lifetimes. While our genomes bear the marks of selection resulting from co-evolution with pathogens [25–32], fortunately for pathogens of vertebrates, the somatic mutation pivotal to the generation of adaptive immune responses is not heritable, therefore each generation presents itself as naive hosts susceptible to infection. Given the array of strategies pathogens have evolved to deal with host immune responses, it is clear that this has selected for pathogens able to cope with immune assault.

Antigenic variation has convergently evolved in many pathogens as a strategy allowing infection of hosts with prior exposure and for infections to persist longer (and in some cases to become chronic). Antigenic variation can be defined as the ability of pathogens to vary the antigens it exposes to the host, by differential expression of antigens belonging to the same family. The molecular mechanisms that govern this variation can differ; from recombination-driven DNA inversion [33–38], gene conversion [39,40], through to epigenetic factors such as subnuclear localization and histone acetylation, and Dam methylation [41–43]. Another strategy evolved to counter the immune response is the ability to directly modulate host immune responses via pathogen-encoded homologues of immune modulators such as Fc receptors and cytokines (reviewed elsewhere [44–46]).

Not only are the generative mechanisms that drive genetic variation pivotal to the evolution of virulence, the expression of these changes is equally important (see Box 1). Selection is thought to favour haploidy in pathogens and diploidy in hosts [47], suggesting that limiting the array of antigens exposed to the host during infection is a driving force for the pathogen during infection. Obviously, the benefit of haploidy is the immediate phenotypic expression of genetic variations; the consequent benefits (or costs) of these mutations through selection within the host will determine the success of these changes. Diploidy in pathogens would be expected to buffer the phenotypic expression of genetic variation, thereby slowing pathogen evolution.

**Box 1. Making Use of Morons**

Morons are genes located between two genes whose homologues are adjacent in related phage [62]. These genes have no direct role in the lytic or lysogenic developmental cycles of the phage, but rather serve as fitness factors for the lysogen via the advantage they afford to the bacterial host. By contributing to the survival of their bacterial host, morons are positively selected. In many ways, virulence genes associated with all mobile genetic elements that are not required for the process of element mobilisation and/or replication could be considered analogous to phage morons.

It was clear from early work on bacterial pathogens such as *Salmonella* and Escherichia coli that genes required for particular biochemical pathways tend to cluster together [63,64]. This clustering of genes of related function into operons, both essential and non-essential, has been argued to be formed by horizontal gene transfer [65] or by co-regulation [66], but what is apparent is that once gene clusters have formed, they can be moved as a unit by horizontal gene transfer between pathogens. This clustering would facilitate the utilization by the host of the functions encoded by the newly acquired DNA.

Barriers exist to the use of horizontally acquired genes following their acquisition. If the genes transferred confer no added fitness, they will likely be lost by random mutation or deletion. Likewise, if the transferred genes reduce the fitness of the new host, they will be lost in the competition with non-converted bacteria. Since the organism into which gene transfer has occurred possesses a biochemical network that has itself been honed and balanced by evolution, the question of how host organisms take advantage of these morons becomes a pivotal one to the evolution of virulence.

Horizontally transferred regions may encode regulators that control the expression of genes within the transferred DNA [67]. However, this does not automatically result in appropriate contextual expression in the regulatory network of a cell. A possible solution to this problem would be to co-opt ancestral regulatory mechanisms already present in the genome of the recipient in order to achieve a profitable expression pattern. Evidence for such occurrences are provided in several bacterial pathogens that incorporate horizontally acquired genes into their virulence program, such as utilization of the OmpR/EnvZ and PhoP/PhoQ two-component regulatory systems by *Salmonella* for coordination of its two type III secretion systems required for pathogenesis [68–70]. Ancestral transcriptional repressors have also been commandeered, such as H-NS [71], YmoA [72,73], Hha [74], and YdgT [75] to fine-tune the expression of virulence factors contained within HGT regions. That these co-opted regulators are as important as the presence of virulence factors themselves is highlighted by the attenuated phenotype of mutants lacking proper regulation.

The utilization by a pathogen of newly recruited virulence factors sometimes necessitates the presence of a delivery system competent to localise the molecule appropriately. One way bacterial pathogens have solved such a problem is to evolve a protein delivery channel on their surface, allowing for translocation of proteins (termed effectors) directly into target host cells. This system is usually encoded within a single locus containing a set of operons. Once this system is in place, new effectors can be added by horizontal transfer in a plug-and-play manner once appropriate regulation and targeting of effectors to the type III system is achieved. The phage protein SopE, associated with the *Salmonella* prophage SopEΦ [76] can be moved by lysogeny into various strains and secreted by the type III secretion system. Another phage moron, called GogB, associated with the Gifsy-1 prophage in some strains of *Salmonella enterica,* can be experimentally transferred to enteropathogenic E. coli as a discrete module where it immediately integrates into the EPEC type III secretion system at the level of co-regulation, targeting, and secretion [77].

The acquisition of appropriate regulation by horizontally transferred fitness factors, such as type III effectors, is as pivotal to the evolution of virulence as the acquisition of the virulence factors themselves. While an important long-standing question has been the unknown origin of horizontally acquired virulence factors, an equally important question is how regulatory elements become installed to result in effective expression.

Amongst the mechanisms of change at the simplest level are point mutation, base substitution, and recombination. Consequently, the rate by which a pathogen is capable of change is encoded in the pathogen's genome (by virtue of the error rates of the molecules and enzymes governing these processes). This simple but important concept infers that the rates of change are themselves subject to natural selection (i.e., that evolvability itself is subject to natural selection) [48]. Within the framework of evolvability being a selectable trait, it is fascinating to see that while mutations in the molecules that govern the repair of mutations (resulting in a hypermutable state) are rare in vitro [49] and deleterious [50] they appear with higher frequencies in hospital environments [51,52], suggesting that genetic flexibility is advantageous within hosts to maintain pathogen fitness [53].

It has been clear for some time that different organisms have different baseline mutation rates [54], but more recently it has become apparent that mutation rates are not uniformly distributed across an organism's genome; for instance, the complementarity determining regions of immunoglobulin exhibit higher mutation rates than those in framework regions [55]. Likewise, pathogens appear to exhibit higher mutation rates at regions of their genomes involved in antigenic drift [56]. A second pivotal point is that pathogen mutation rates appear higher in vivo than in vitro [57], suggesting that the interaction of pathogen and host itself facilitates the generation of variation. For bacterial pathogens, the frequency of horizontal gene transfer (central to the acquisition of virulence determinants in bacterial pathogens) appears higher in vivo than in vitro (Box 2), suggesting that host–pathogen interaction is not only involved in the selection for pathogen fitness factors, but may directly contribute to the generation and dissemination of these fitness determinants.

In bacteria, pathogenicity-associated genes commonly reside in mobile genetic elements (or their remnants) within the genomes of pathogens. These mobile genetic elements include plasmids, phage, conjugative transposons, transposons, and integrons, all of which can be transferred between genomes, generating variation. This horizontal gene transfer (which has been demonstrated experimentally, see Box 2) is thought to lead to the acquisition of new traits upon transfer to new hosts, and the prevalence of genomic islands (and genomic islets) suggests this transfer is widespread [58]. Other pathogens, such as *Plasmodium* and *Trypanosoma,* benefit from a sexual cycle and the recombination between genomes it provides [59,60]. Likewise, viruses that infect the same cell can recombine genomes, often resulting in the antigenic shift associated with virulent strains. Gene acquisition such as this has profound effects by allowing pathogens to evolve rapidly to overcome selective pressures.

**Box 2. Has the Struggle between Host Resistance and Mobile Genetic Element Given Rise to Virulence?**

William Hayes, who pioneered work on conjugative gene transfer (convergent with the work of the Lederbergs and Cavalli [78]), has suggested that with regard to bacterial genomes, it is no longer possible for us to ‘draw a firm line of demarcation between chromosomal and cytoplasmic determinants, between viral and non-viral elements, or even between viral and bacterial genes' [79]. Already, genetics had made it clear that genes may spread throughout different bacterial species via natural transformation, transduction, and conjugation.

Over the last 20-odd years, it has become even clearer that bacterial pathogens contain an amazing array of horizontally acquired DNA. Amongst these regions of DNA are not only selfish DNA molecules (which contain only the elements required for mobilisation and do not contribute to the fitness of the host) such as IS elements (which some might argue could be the simplest form of life), but also transposons, plasmids, prophages, and regions known as genomic islands (some of which may also be regarded as selfish DNA); all of which can harbour virulence genes.

Since these regions of DNA have been acquired horizontally, their presence infers that within these regions are one or more genes that increase the fitness of bacteria harbouring this region of landed foreign DNA. Thus the advantage conferred by these genes has presumably selected for the spread of the mobile regions of DNA they reside in. The sheer volume of horizontal gene transfer inferred from sequence gazing suggests that this process is pivotal to the evolution of virulence, but an important unanswered question is what facilitates this spread at the molecular level.

Of course, it is in part the immune response to pathogens that provides the selective pressure for the acquisition of virulence factors that facilitate colonisation in the face of this immune assault. But might the immune response promote the dissemination of mobile genetic elements and the virulence factors they contain in a more direct sense, by stimulating their mobilization? Were this to be the case, mobile genetic elements would appear to have not only have co-opted bacteria but also the parasitised host in their struggle for existence. As a consequence, disease and the evolution of bacterial pathogenicity may be an unfortunate but necessary result of the battle between parasitised host and mobile genetic elements.

Work comparing DNA mutation rates of pathogens suggests mutation rates are higher when pathogens are within a host context [57]; could this be indicative of the stress of dealing with immune assault? Phage that encode virulence determinants such as Shiga-toxin have been shown to lysogenise commensal *E.coli* in vivo [80]. That such transfer events occur at a much higher frequency in vivo than in vitro [81] suggests there is indeed something about the host that promotes the spread of phage containing fitness factors. What are the host agents that promote the movement of mobile genetic elements?

One candidate is reactive oxygen species produced by phagocytes; which has been shown to induce a lytic stage in both E. coli and *Salmonella* prophages [82,83]. From the bacterial side, the bacterial SOS response to DNA damage has been implicated in the induction of phage transfer [84–87]. In addition, this SOS response also facilitates horizontal transfer of conjugative elements [88], suggesting mobile genetic elements have co-opted a global response to DNA damage to regulate their escape from damaged (bacterial) hosts. The ability of agents that induce the SOS response, such as reactive oxygen species, mitomycin C, and antibiotics (including fluoroquinolone antibiotics and DHFR inhibitors), to facilitate conjugative transfer suggests that transfer may be promoted by any agent that induces the SOS response.

It is possible that other components of the innate immune system promote mobile genetic element transfer in vivo. Defective cell wall synthesis (such as that induced by B-lactam antibiotics) has been shown to initiate the SOS response, allowing bacteria to survive longer in the presence of an antibiotic [89]. Given that many antimicrobial peptides, molecules central to the innate immune system, inhibit cell wall synthesis or impair bacterial cell septum formation [90–93], it is possible that antimicrobial peptides also induce the bacterial SOS response. As a consequence, antimicrobial peptides may facilitate the transfer of mobile genetic elements between bacteria, although this important possibility remains unaddressed in any model system. The availability of mice deficient in particular antimicrobial peptides should allow dissection of the importance of these molecules in mobilisation of transferable virulence genes.

The innate immune response has selected for the dissemination of mobile genetic elements by virtue of providing selection for the virulence genes contained within them (i.e., the advantage these genes afford to the pathogen). If antimicrobial peptides were able to promote the transfer of mobile genetic elements in vivo, it may be that mobile genetic elements not only sense (indirectly) the innate immune response [94] but utilise it towards their own end, to spread amongst the ranks of pathogen that struggle for existence.

## Applications and Future Challenges

The processes of selection and evolution of virulence traits is a continuum, influenced by both deterministic and stochastic events that are interminable. However, one thing is clear; the evolution of hosts and the pathogens that exploit them are inexorably linked. The genetic composition of individual hosts shape and fine-tune pathogen virulence, and the reciprocal genetic sculpting process is also true. Harrowing for us, bacterial pathogens possess consummate skill at testing the waters, using genetic elements acquired from external reservoirs, followed by diversification in various ecological niches that ultimately maximize their propagation and transmission. Intense effort is underway to learn what portions of DNA harbour the salient features of host defences and pathogen virulence, and accomplishments towards this goal will continue as genomics and bioinformatics fertilise the field of evolutionary biology.

Increasing knowledge of how pathogen virulence traits are acquired and selected in various populations is consequential for several reasons. In practical terms relating to global human health, it is apropos to bring to bear information concerning the evolution and maintenance of pathogen virulence traits to applied ends. Policies governing infection control programs and intervention scenarios ought to be instructed by evidence-based data on how pathogens persist in human populations, how such infections are spread amongst the population, and which animal or environmental reservoirs might contribute to the spread. Novel approaches to quantifying the spread of infection based on surveillance, contact tracing [61], and geographical patterns of transmission are being implemented, taking into account stochastic events during early stages of pathogen transmission that are difficult to model. The incorporation of genomic data into such models should be a benefit to pathogen surveillance efforts and risk assessment strategies. This presupposes that quantitative and qualitative measures of risk associated with certain genetic elements within pathogens are developed, and indeed the genomics era has facilitated this.

The challenge for future research is to take the basic concepts of evolutionary biology beyond the selection and maintenance of virulence traits and apply these principles towards logical predictions of how certain pathogens will behave in a population over evolutionary time. Progress towards this end requires collaboration among evolutionary biologists, epidemiologists, social scientists, and basic scientists. Definitive answers to how and where pathogens acquire new genetic material, how these genes are expressed and selected for, and the relative risks associated with certain genetic elements will allow a more complete comprehension of the evolution of infectious diseases.

## Abbreviations

DHFR: dihydrofolate reductase
EPEC: enteropathogenic Escherichia coli
IS: insertion sequence
